# Prevalence, incidence, morbidity and mortality rates of COPD in Saudi Arabia: Trends in burden of COPD from 1990 to 2019

**DOI:** 10.1371/journal.pone.0268772

**Published:** 2022-05-19

**Authors:** Jaber S. Alqahtani

**Affiliations:** 1 Department of Respiratory Care, Prince Sultan Military College of Health Sciences, Dammam, Saudi Arabia; 2 Head of Scientific Research and Publication Department, Vice Deanship of Postgraduate Studies and Research, Prince Sultan Military College of Health Sciences, Dammam, Saudi Arabia; Taif University, SAUDI ARABIA

## Abstract

**Background:**

The available data to determine the chronic obstructive pulmonary disease (COPD) burden in Saudi Arabia are scarce. Therefore, this study closely examines and tracks the trends of the COPD burden in Saudi Arabia from 1990 to 2019 using the dataset of the Global Burden of Disease (GBD) 2019.

**Methods:**

This study used the GBD 2019 dataset to analyse the COPD prevalence, incidence, morbidity and mortality rates in the Saudi Arabian population from 1990 to 2019, stratified by sex and age. The age-standardised rate was used to determine the prevalence, incidence, years of life lost (YLLs), years lived with disability (YLDs), disability-adjusted life years (DALYs) and deaths.

**Results:**

In 2019, an estimated 434,560.64 people (95% Uncertainty Interval (UI) 396,011.72–473,596.71) had COPD in Saudi Arabia, corresponding to an increase of 329.82% compared with the number of diagnosed people in 1990 [101,104.05 (95% UI 91,334.4–111,223.91)]. The prevalence rate of COPD increased by 49%, from 1,381.26 (1,285.35–1,484.96) cases per 100,000 in 1990 to 2,053.04 (1918.06–2194.29) cases per 100,000 in 2019, and this trend was higher in males than females. The incidence rate of COPD in 2019 was 145.06 (136.62–154.76) new cases per 100,000, representing an increase of 43.4% from the 1990 incidence rate [101.18 (95.27–107.86)]. In 2019, the DALYs rate was 508.15 (95% UI 434.85–581.58) per 100,000 population. This was higher in males than females, with a 14.12% increase among males. In 2019, YLLs contributed to 63.6% of DALYs due to COPD. The death rate due to COPD was 19.6 (95% UI 15.94–23.39) deaths per 100 000 in 2019, indicating a decrease of 41.44% compared with the death rate in 1990 [33.55 deaths per 100 000 (95% UI 25.13–47.69)]. In 2019, COPD deaths accounted for 1.65% (1.39–1.88) of the total of deaths in Saudi Arabia and 57% of all deaths caused by chronic respiratory diseases.

**Conclusion:**

Over the period 1990–2019, the prevalence and incidence of COPD in Saudi Arabia have been steadily rising. Even though COPD morbidity and death rates have been decreasing, they remain higher in men and older age. The holistic assessment and interventions with careful attention to optimising the community-based primary care management, such as screening for early diagnosis, smoking cessation programs and pulmonary rehabilitation, are likely to be the most successful strategies to reduce the burden of COPD in Saudi Arabia.

## Introduction

Chronic obstructive pulmonary disease (COPD) is one of the most common non-communicable diseases with the potential to be life-threatening. It is caused primarily by tobacco smoking and air pollution [1]. According to the Global Initiative for Chronic Obstructive Lung Disease (GOLD), COPD is a common, treatable and curable disease [2]. COPD is now the third major cause of mortality globally and is expected to reach the top of the list over the next decade [2]. In 2019, there were an estimated 455 million cases of COPD and 3.9 million deaths globally [3]. COPD is a serious public health problem that has a detrimental financial effect. The worldwide cost of COPD in 2010 was $ 2.1 trillion, which is estimated to climb to $4.8 trillion by the year 2030 [4]. This is a conservative estimate because COPD continues underdiagnosed. Furthermore, the long list of comorbidities, such as cardiovascular disease, lung cancer and mental health associated with COPD, would place an extra burden on the healthcare system [5–7].

In the Middle East and particularly in the Gulf Cooperation Council nations, COPD remains underdiagnosed and underestimated [8]. Saudi Arabia is considered the largest country in the Middle East region with a population of 35.6 million people as of 2022, based on Worldometer data [9]. Several studies conducted in Saudi Arabia over the last three decades reveal that smoking is on the rise, especially among men and women in their twenties [10–12]. A representative study conducted in 2013 found that the overall prevalence of smoking was 12.2%, and men were more likely to smoke than women (21.5% vs. 1.1%) [13]. According to the BREATH survey conducted in MENA nations, Saudi Arabia has a smoking prevalence of 16% for cigarettes alone, 5% for water pipes alone, 4% for water pipes and cigarettes together, and 25% for all types of smoking [14]. In Saudi Arabia, the age and gender-adjusted prevalence of COPD-related symptoms, defined as chronic productive cough and/or dyspnoea, was 14.3% [15]. Yet, according to the epidemiological definition of COPD, only 2.4% of the participants met the criteria (symptoms or diagnosis and cigarette smoking 10 pack-years), while 2.8% were diagnosed with chronic bronchitis according to the GOLD diagnostic criteria [15]. In a study with 501 smokers in three major Saudi cities, the prevalence of COPD was 14.2% [16]. Additionally, many individuals in Saudi Arabia, also report a history of tuberculosis, persistent asthma, and respiratory-tract infections throughout infancy, which are all considered risk factors for COPD [17]. Further, non-smoking variables, such as biomass fuel, dusts, gases, and outdoor air pollution, are also commonly contributing to the increased burden of COPD in Saudi Arabia [18]. There are just a few pulmonary rehabilitation programs that are not extensively used in Saudi Arabia, owing to a lack of promotion and workforce competence [19]. Unavailability or underutilisation of such services would exacerbate the kingdom’s COPD burden.

Despite the high global burden of COPD, the available epidemiological data about COPD in Saudi Arabia are limited and do not fully report the age-standardised rates of prevalence, incidence, morbidity and mortality of COPD that stratified by gender. For more than 200 countries and regions, the Global Burden of Disease (GBD) 2019 presents the most up-to-date evaluation of the descriptive epidemiology of a mutually exclusive and collectively complete list of diseases and injuries from 1990 to 2019 [3]. For government policymakers and healthcare professionals, it is critical to understand the national trends of prevalence, incidence, morbidity and mortality associated with COPD that have great public health and socioeconomic burden. Therefore, this study aims to track trends and present the health burden of COPD in Saudi Arabia using the GBD 2019 dataset.

## Methods

This study made use of the publicly accessible data from the World Health Organisation (WHO) and the GBD repository of the Institute for Health Metrics and Evaluation [20]. This paper explores the recent GBD 2019 trends of the prevalence and incidence of COPD in Saudi Arabia, as well as the trends in disability-adjusted life years (DALYs) and mortality ascribed to COPD, over the period 1990–2019, stratified by sex and age. The GBD 2019 study’s methodology and data-collection processes have already been described in detail [3, 21]. Briefly, multiple relevant data sources were identified for each disease or injury in the GBD-estimation process, including censuses, household surveys, civil registrations and vital statistics, disease registries, health care utilisation, air pollution monitors, satellite imaging and disease notifications.

Nonfatal and fatal estimates for COPD were also based on thorough evaluations of published articles, unpublished reports, and survey data from the GBD’s Global Health Data Exchange repository in 2019. Because of the lack of a primary source of data from Saudi Arabia, the COPD covariates that predicted morbidity and mortality were used. These covariates were the cumulative cigarette smoking for the last years, second-hand smoke, indoor and outdoor air pollution, use of biomass for cooking or heating and occupational exposure. The surveys and research papers published over the last three decades were used to compile data for the calculation of the COPD burden. The GBD 2019 Data Input Sources Tool for Saudi Arabia contains all of the relevant papers and publications [22].

Years of life lost (YLLs) were calculated by multiplying the number of COPD-caused deaths by the remaining life expectancy derived from the GBD standard life table [23]. Similarly, the number of years lived with disability (YLDs) was determined by multiplying the prevalence by the disability weight of the person with the impairment. Subsequently, the total number of YLLs and YLDs was used to calculate DALYs [3, 24]. The author calculated the trend percentage of our outcomes to compare each measure over time of a baseline year. The age-standardised rate was used to adjust for differences in the age distribution of the population for more representative outcomes. Stratification by sex was applied to compare the trends between male and female for all the previous measures. The author reported 95% uncertainty intervals (UIs) for all estimations, with UIs reflecting the 2.5^th^ and 97.5^th^ percentiles of a 1000-draw distribution at each stage. The GBD data have been de-identified and made public, thus, this study is exempt from evaluation by the institutional ethics board. Statistical Package for the Social Sciences (SPSS) version 28 was used to analyse the data (IBM Corp. Armonk, New York, USA). The GBD data can be access via http://ghdx.healthdata.org/.

## Results

### Prevalence of COPD in Saudi Arabia

**Fig 1:**
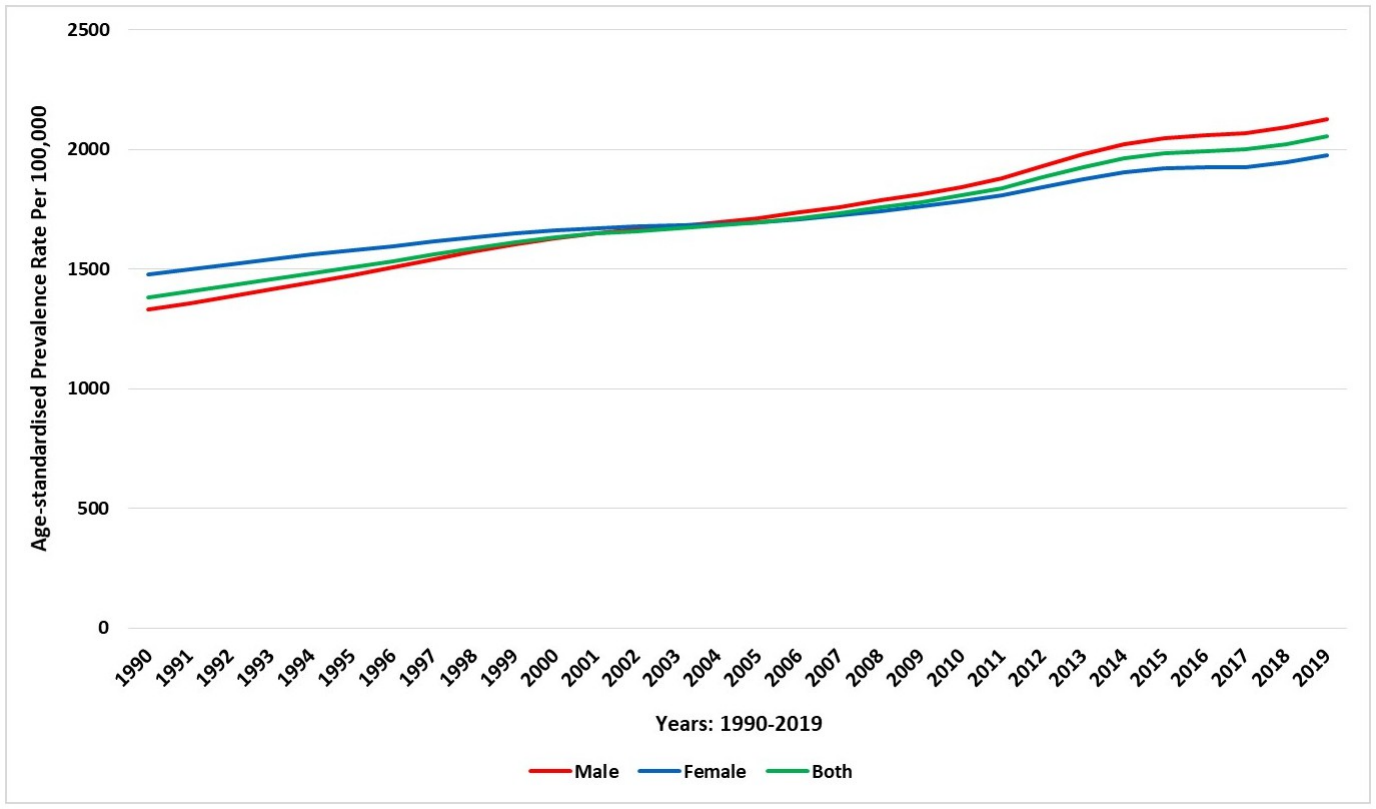
Trends in the age-standardised prevalence rate of COPD during 1990–2019 in Saudi Arabia, stratified by gender.

**Fig 2:**
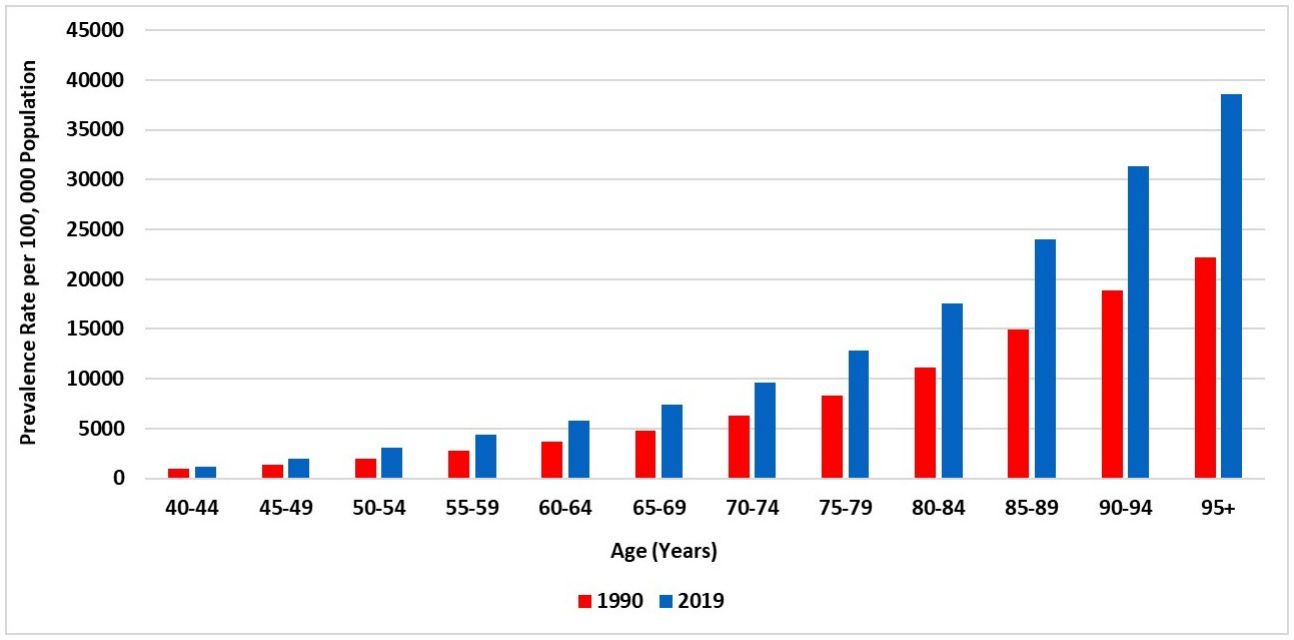
The prevalence rate of COPD in Saudi Arabia (1990 and 2019), stratified by age.

In 2019, an estimated 434,560.64 people (95% UI 396,011.72–473,596.71) had COPD in Saudi Arabia, corresponding to an increase of 329.82% compared with the diagnosed population in 1990 [101,104.05 (95% UI 91,334.4–111,223.91)]. The age-standardised prevalence rate of COPD in 1990 was 1,381.26 (1,285.35–1484.96) cases per 100,000 and 2,053.04 (1,918.06–2,194.29) cases per 100,000 in 2019, demonstrating an increase of 49%. In 2019, the age-standardised prevalence rate of COPD for males [2,123.71 (1,980.21–2,283.64)] was 7.49% higher than that for females 1,975.70 (1,829.1–2,120.23). During the study period, there was an increase of 33.8% (1,477.19 vs 1.975.70) and 37.4% (1.328.79 vs 2.123.71) in the age-standardised prevalence rate of COPD in females and males, respectively. Fig 1 shows the trend in the age-standardised prevalence rate of COPD during 1990–2019 by gender in Saudi Arabia. Between 1990 and 2019, the prevalence rate of COPD in Saudi Arabia has increased with increasing population age (Fig 2).

### Incidence of COPD in Saudi Arabia

**Fig 3:**
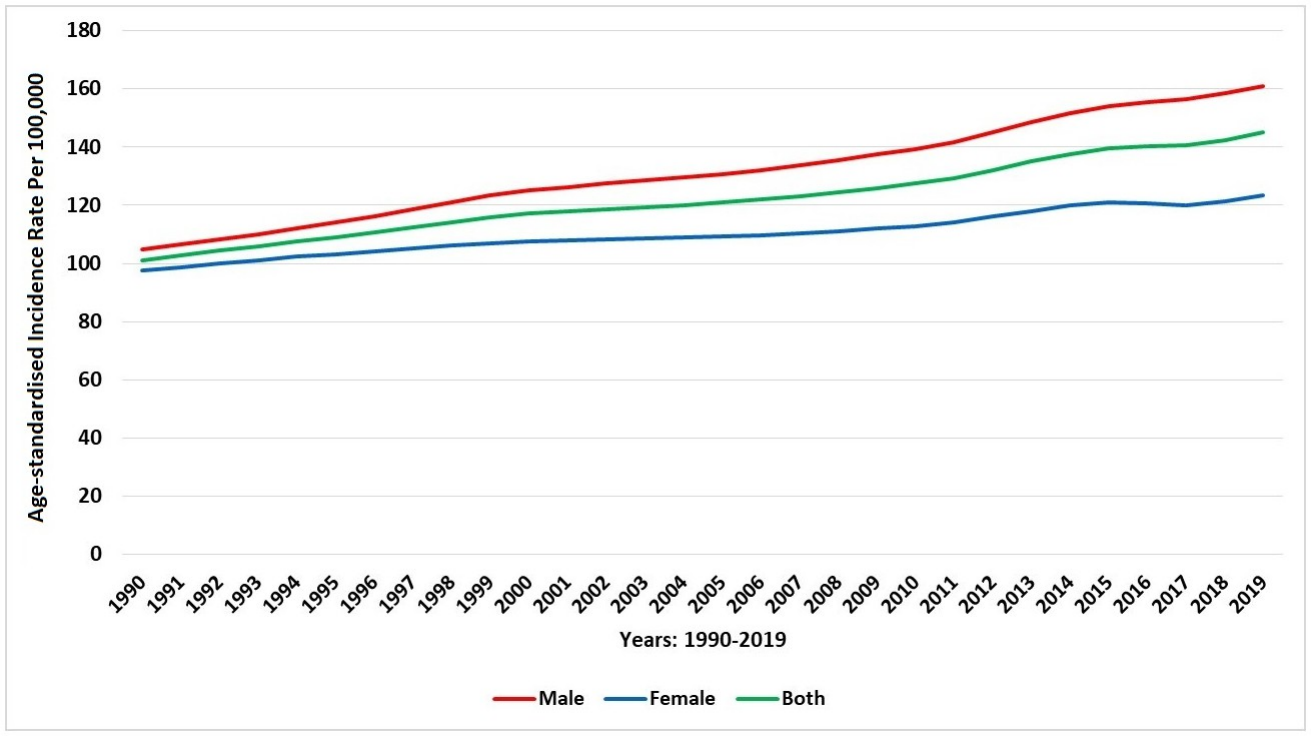
Trends in the age-standardised incidence rate of COPD during 1990–2019 in Saudi Arabia, stratified by gender.

**Fig 4:**
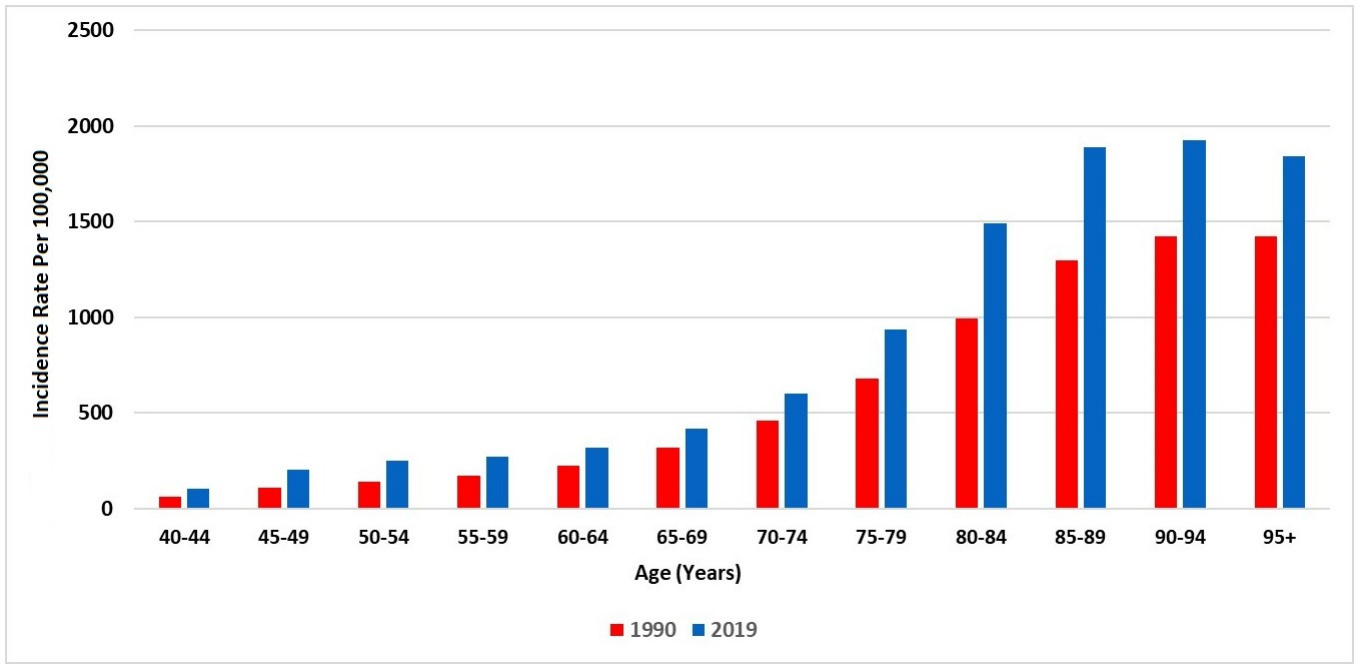
The incidence rate of COPD in Saudi Arabia (1990 and 2019), stratified by age.

Number of new COPD cases in 2019 was estimated to be 31,296.94 (95% UI 29,020.68–33,630.52), indicating an increase of 323.6%, compared with number of new COPD cases in 1990, 7388.25 (95% UI 6909.6–7898.9). The age-standardised incidence rate of COPD in 1990 was 101.18 (95.27–107.86) new cases per 100 000 and 145.06 (136.62–154.76) new cases per 100 000 in 2019, representing an increase of 43.4%. There was stagnant increase from 1990 to 2019 in the incidence rate of COPD in both males (104.65 vs 161.05, 54%) and females (97.49 vs 123.50, 27%). Fig 3 presents the trend of age-standardised incidence rate of COPD during 1990–2019 in Saudi Arabia, stratified by sex. Between 1990 and 2019, the incidence rate of COPD in Saudi Arabia has risen as the population has become older (Fig 4).

### Morbidity caused by COPD in Saudi Arabia

Overall, there were 91,981.72 DALYs (95% UI 77,924.92–107,172.03) due to COPD in 2019, representing an increase of 135.7% since 1990 [39,029.28 DALYs (30,653.07–52,587.75)]. In 2019, the overall age-standardised DALYs were 508.15 (95% UI 434.85–581.58) per 100,000 population. For males, DALYs were 534.7 (95% UI 436.88–626.66) per 100,000 population and for females, they were 468.54 (95% UI 376.27–571.31) per 100,000 population. This represents an increase of 14.12% in the age-standardised DALYs among males compared to females.

**Fig 5:**
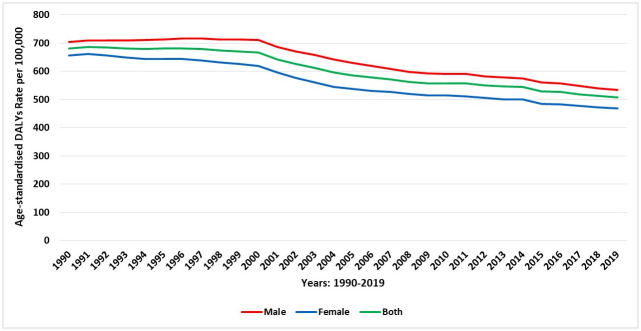
Trends in the age-standardised DALYs rate of COPD during 1990–2019 in Saudi Arabia, stratified by gender.

**Fig 6:**
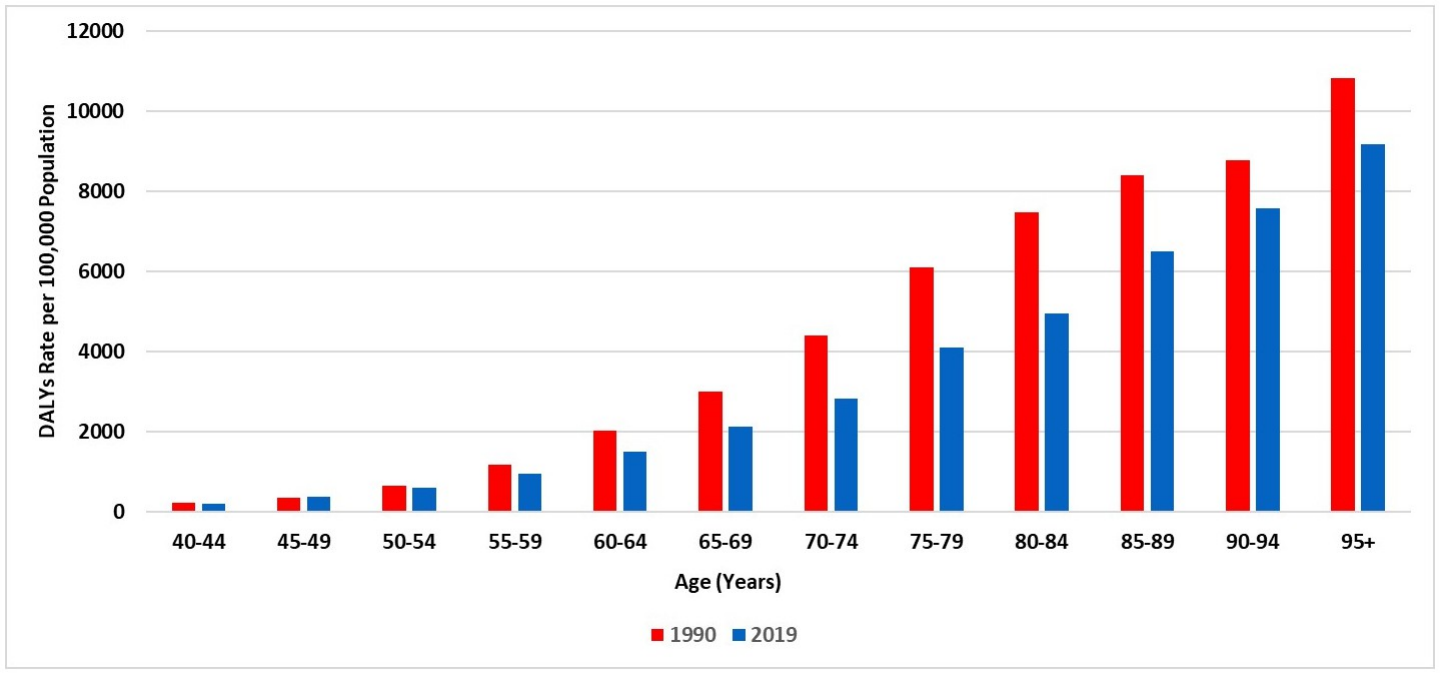
The DALYs rate of COPD in Saudi Arabia (1990 and 2019), stratified by age.

In 2019, the age-standardised YLLs was 323.12 (95% UI 258.02–389.43) per 100,000 population, which contributed to approximately 63.6% of DALYs due to COPD. Likewise, the overall YLDs was 185.04 (95% UI 150.4–215.13) per 100,000 population in 2019, contributing to 36.4% of the morbidity caused by COPD. In 2019, YLDs was higher among females [age-standardised YLDs: 192.82 (95% UI 155.35–224.28) per 100,000 population] than for the males [181.15 (95% UI 144.81–216.31) per 100,000 population]. Fig 5 shows the trend of DALYs stratified by sex over the last 30 years. Across different age groups, there was a trend of reduced DALYs in 2019 compared to 1990, but the rate of DALYs increased along with the increased age (Fig 6).

### Mortality caused by COPD in Saudi Arabia

**Fig 7:**
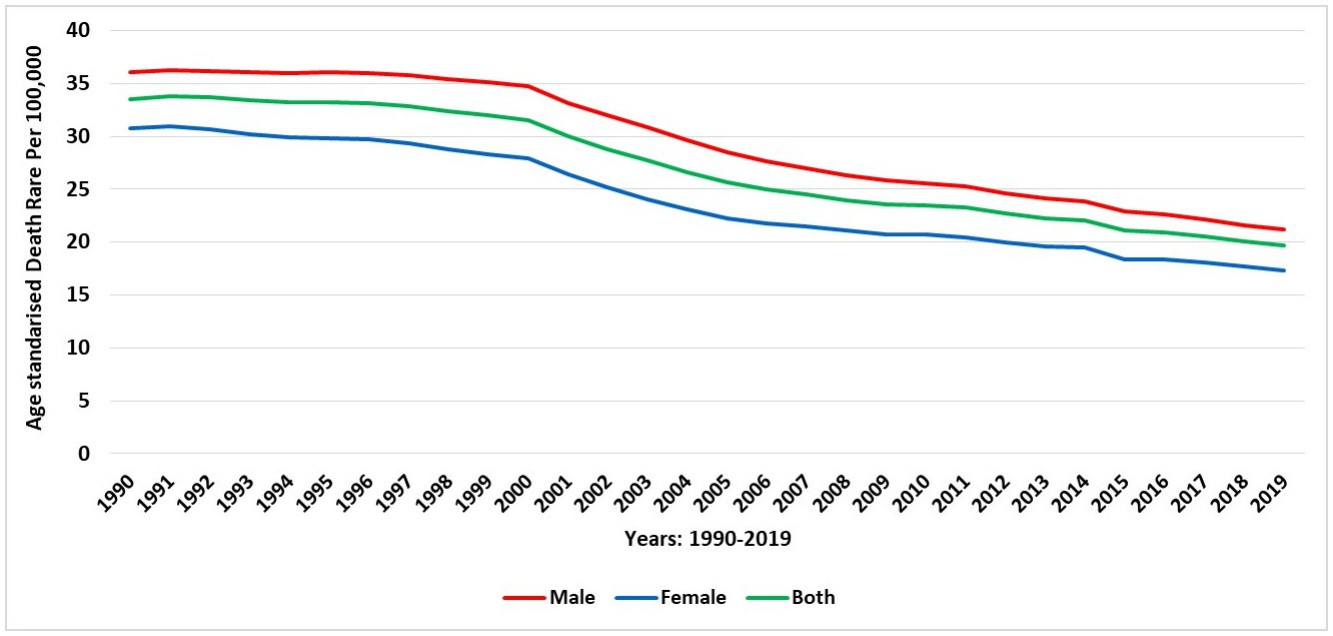
Trends in the age-standardised death rate of COPD during 1990–2019 in Saudi Arabia, stratified by gender.

**Fig 8:**
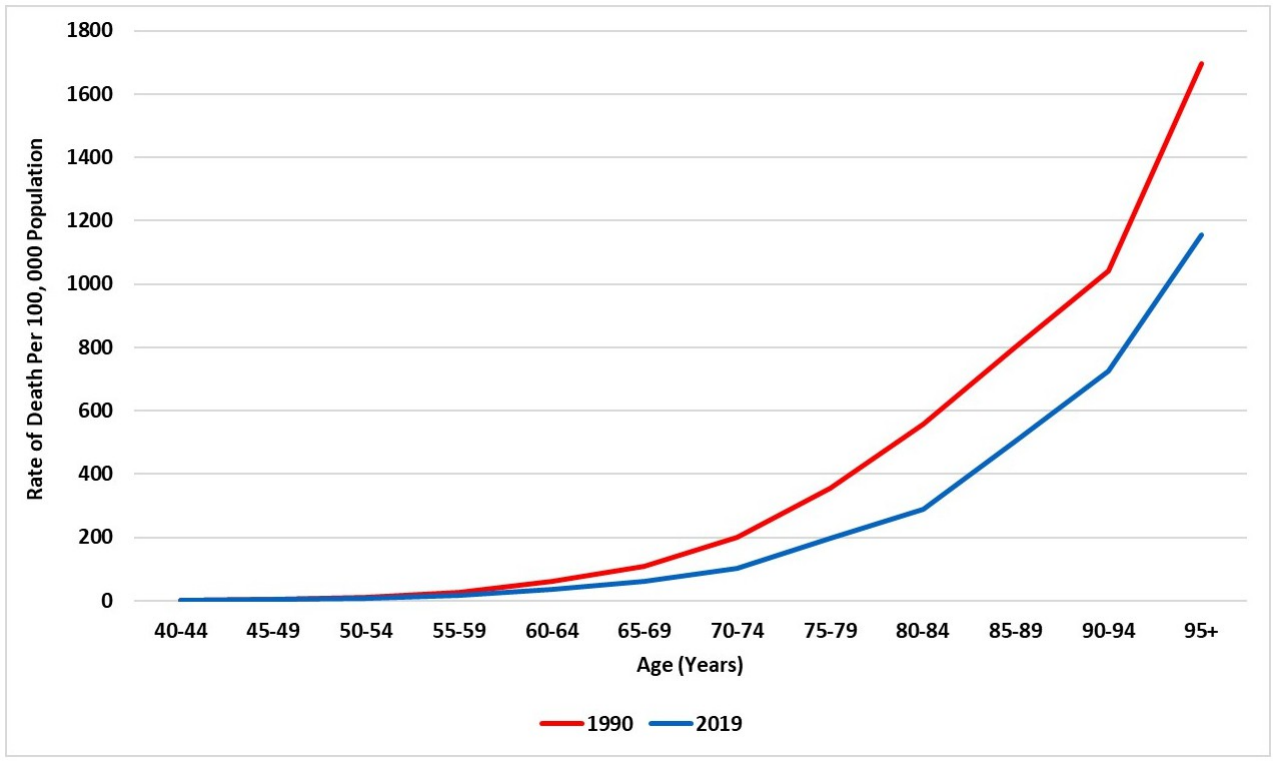
The death rate of COPD in Saudi Arabia (1990 and 2019), stratified by age.

There were 2,119.22 (95% UI 1685.24–2576.13) deaths due to COPD in 2019, revealing an increase of 47.91% since 1990 [1432.73 (95% UI 1072.52–2089.28)]. These deaths, in total, accounted for 1.65% (1.39–1.88) of the total all-cause deaths in Saudi Arabia and corresponded to 57% of the deaths caused by chronic respiratory diseases, such as asthma and interstitial lung disease. In 2019, the age-standardised death rate due to COPD was 19.6 (95% UI 15.94–23.39) deaths per 100,000, indicating a decrease of 41.44% compared with the death rate in 1990 [33.55 (95% UI 25.13–47.69) deaths per 100,000]. During the study period, the age-standardised death rate due to COPD reduced for both males (36.05 vs. 21.15; a decrease of 41.33%) and females (30.72 vs. 17.34; a decrease of 43.56%), but the drop was considerably higher among females when compared with males. Fig 7 demonstrates the trends of mortality rates over the period 1990–2019 stratified by sex. Overall, the rates of death due to COPD were reduced across all age categories from 1990 to 2019, although the mortality rate increased along with increased age (Fig 8).

## Discussion

To the author’s knowledge, this is the first study to thoroughly investigate the COPD burden in Saudi Arabia using the GBD database that includes prevalence, incidence, morbidity and mortality rates during 1990–2019. The age-standardised prevalence and incidence rates in Saudi Arabia have rapidly increased from 1990 to 2019, by 49% and 43.4%, respectively. These rates were higher in males than females and increased in tandem with the population’s aging. A steady drop in the age-standardised morbidity and death rates was found throughout the same period, with the decline being greater among females than males. As the Saudi population’s age increases, the rates of morbidity and death consistently rise. Management of respiratory conditions in Saudi Arabia may be affected by factors such as health care costs, religious beliefs and cultural practices, as well as a failure to follow evidence-based treatment standards [18]. According to the Saudi Ministry of Health, respiratory disorders were Saudi Arabia’s fifth highest cause of death in 2014 [25]. Such data highlight the need for developing effective strategies to mitigate this burden.

Saudi Arabia’s rate of COPD prevalence in 2019 was 2,053.04 (1,918.06–2,194.29) cases per 100,000, demonstrating an increase of 49% compared to 1990. This is lower than the global prevalence rate of 2,639.2 (2,492.17–2,796.14) per 100,000 population [3]. The prevalence rate is also lower than those of high-income countries such as the United Kingdom with 3,902.15 (3,678.51–4,125.74) and the United States with 3,668.54 (3,514.64–3,805.41) cases per 100,000 population [3]. The increased prevalence of COPD in Saudi Arabia can be justified by the increased rate of smoking among adults, the use of water pipes, biomass fuel exposure, outdoor air pollution and the increasing age of the population [26]. As a result of increasing population aging, incidence, morbidity and mortality are increased, highlighting the importance of early COPD screening and diagnosis [27].

The age-standardised incidence rate of COPD in Saudi Arabia was increased by 43.4% from 1990 to 2019 [101.18 (95.27–107.86) vs. 145.06 (136.62–154.76) new cases per 100,000, respectively] owing to the increase of the population’s age. Such an increase might be due to improvements in COPD health care expertise that allow the early detection and treatment of the disease, hence, reducing the risk of mortality and disability. However, this rate is still lower than the global incidence rate of 200.49 (188.63–212.57), which indicates that intense COPD screening is necessary to unravel possible discrepancies caused by inaccurate and insufficient data collection. Tobacco-control measures and attention to decrease the exposure to indoor and outdoor air pollution are still needed to lower the risk of developing COPD [3].

The trends of DALYs and mortality categorised by sex decreased in the Saudi population over the last 30 years, but both rates increased along with the increased lifespan. As a result, the burden of COPD in Saudi Arabia is estimated to dramatically rise as the population ages, especially in males that have a higher prevalence and incidence of COPD than females. Indeed, Saudi Arabia had lower rates of DALYs (508.15 vs. 926.8 per 100,000 population) and mortality (19.6 vs. 42.52 deaths per 100,000) compared to the global rates. The reason for such a decrease could be related to the younger age of the Saudi population, with over half of the population being 35 years old or younger [28]. However, the younger Saudi population is more likely to be overweight and sedentary to older portions of the population, and adheres to poor eating habits [29]. One of the aims of the Saudi 2030 vision is to reduce the fertility rate and increase life expectancy. The population above 60 is expected to rise from 5.5% in 2020 to 11.0% by 2030 [28]. Therefore, to meet this national goal, there is a need to enhance the quality and efficiency of healthcare services, as well as encourage prevention against risk factors for COPD.

In 2019, deaths attributed to COPD reduced to 1.65% compared to 1990 (1.74%), accounted for 1.65% (1.39–1.88) of the total all-cause deaths in Saudi Arabia and corresponded to 57% of all deaths caused by chronic respiratory diseases, such as asthma and interstitial lung disease. This reduction was consistent with the WHO’s report on non-communicable disease mortality [30]. The Saudi Arabian Ministry of Health invested in healthcare services, recognising some key concerns such as access and health staff development [28]. In addition, they collaborated with international organisations to address the increased prevalence of non-communicable diseases and their risk factors [31]. Furthermore, the Saudi Initiative for Chronic Airway Diseases panel has established the first guidelines for COPD to offer tools for the diagnosis and thorough management of COPD, tailored to the specific needs of the local population [32]. Overall, these national initiatives might have contributed to the decreased rates of morbidity and mortality found in this study. Nevertheless, in the Middle East and Africa, there are significant unmet needs in the care of COPD that should be addressed in the future [33].

For those people living with COPD, the coronavirus disease 2019 (COVID-19) pandemic has been difficult. In spite of the low prevalence of COPD patients in COVID-19 cases, COPD has been linked to higher COVID-19 severity and mortality [34]. As a result, a number of legislative measures were put in place, such as requiring people to wash their hands more often, wearing face masks, and physical distancing to help reduce the risk of infection [35]. According to a recent systematic review and meta-analysis, hospital admissions for COPD exacerbations were reduced by 50% during the COVID-19 pandemic compared to pre-pandemic period [36]. Such findings were likely linked to a decrease in respiratory viral infections that cause exacerbations. Other factors that could contribute to this reduction includes: fear of COVID-19 (increase in medication adherence and behavioural changes), mortality and reduction in pollution [36]. Therefore, COVID-19 pandemic may positively or negatively affect morbidity and mortality of COPD patients in Saudi Arabia. In a recent research prioritisation exercise, patients and clinicians ranked preventing COPD exacerbations as the most important research priority [7]. As of now, there is a lack of studies assessing COVID-19 impact on COPD burden in Saudi Arabia, thus, studies are needed to assess the impact of COVID-19 on the rate of COPD exacerbations, morbidity and mortality.

This study has important clinical and research implications. First, it summarises the burden of COPD in Saudi Arabia and highlights the major trends since 1990. Also, it reveals that the burden of COPD is growing, and public health policy is required to counteract this trend. With the aim of lowering the burden of COPD, community-based primary care management must be established, such as smoking cessation programs, pulmonary rehabilitation and screening for early diagnosis that are both cost-effective and supported by an efficient health monitoring system. Moreover, the burden of COPD and the risk factors it entails may be effectively managed through health education and awareness campaigns targeting the general population, especially the younger population. In 2017, the top 17 health concerns in Saudi Arabia were all related to the environment, including pollution and water contamination, among others [37]. Strategies to mitigate this phenomenon are necessary, such as the effective implementation of renewable energy sources. Finally, research activities to understand the COPD burden in Saudi Arabia are needed because of the lack of statistical data concerning not only the prevalence of COPD but also its severity, the burden on healthcare systems, the economic effect, the social and family views of treatment and occupational risk factors.

This study has some limitations. Because of the unavailability of primary data, the researcher used the GBD database that depends on predictive covariates, which may underestimate the current morbidity and mortality rates. Even if the data were available, they may not have been collected following the recommended definition or measuring technique. However, GBD adheres to “Guidelines for Accurate and Transparent Health Estimates Reporting: The GATHER Statement” that synthesises information from diverse sources to reach reliable findings [38]. For the first time, the data in this study was used to produce the trends of prevalence, incidence, and morbidity and mortality rates of COPD during 1990–2019 in Saudi Arabia, which allow comparison with other countries.

## Conclusion

From 1990 to 2019 in Saudi Arabia, increased age-standardised prevalence and incidence rates of COPD were found; these were higher among males than females. Age-standardised morbidity and mortality rates declined from 1990 to 2019, but these rates were higher among Saudi males than females. As the Saudi Arabian COPD population becomes older, the rates of morbidity and death progressively rise. The holistic assessment and interventions with careful attention to optimising the community-based primary care management, such as screening for early diagnosis, smoking cessation programs and pulmonary rehabilitation, are likely to be the most successful strategies to reduce the burden of COPD in Saudi Arabia.

## References

[1] Eisner MD, Anthonisen N, Coultas D, Kuenzli N, Perez-Padilla R, Postma D, et al. An official American Thoracic Society public policy statement: Novel risk factors and the global burden of chronic obstructive pulmonary disease. Am J Respir Crit Care Med. 2010;182(5):693–718. doi: 10.1164/rccm.200811-1757ST 20802169

[2] Global Initiative for Chronic Obstructive Lung Disease. Global Strategy for the Diagnosis, Management, and Prevention of Chronic Obstructive Pulmonary Disease. 2021 [https://goldcopd.org/wp-content/uploads/2019/11/GOLD-2020-POCKET-GUIDE-FINAL-pgsized-wms.pdf.

[3] Global burden of 369 diseases and injuries in 204 countries and territories, 1990–2019: a systematic analysis for the Global Burden of Disease Study 2019. Lancet. 2020;396(10258):1204–22. doi: 10.1016/S0140-6736(20)30925-9 33069326 PMC7567026

[4] The Global Economic Burden of Non-communicable Diseases 2011 [www.weforum.org/EconomicsOfNCD.

[5] Hurst JR, Siddharthan T. Global Burden of COPD: Prevalence, Patterns, and Trends. Handbook of Global Health. 2020:1–20.

[6] Alqahtani JS, Njoku CM, Bereznicki B, Wimmer BC, Peterson GM, Kinsman L, et al. Risk factors for all-cause hospital readmission following exacerbation of COPD: a systematic review and meta-analysis. Eur Respir Rev. 2020;29(156). doi: 10.1183/16000617.0166-2019 32499306 PMC9488450

[7] Alqahtani JS, Aquilina J, Bafadhel M, Bolton CE, Burgoyne T, Holmes S, et al. Research priorities for exacerbations of COPD. Lancet Respir Med. 2021;9(8):824–6. doi: 10.1016/S2213-2600(21)00227-7 34000234

[8] Idrees M, Koniski ML, Taright S, Shahrour N, Polatli M, Ben Kheder A, et al. Management of chronic obstructive pulmonary disease in the Middle East and North Africa: results of the BREATHE study. Respir Med. 2012;106 Suppl 2:S33–44. 23290703 10.1016/S0954-6111(12)70013-6

[9] Worldometers Data 2022 [https://www.worldometers.info/world-population/saudi-arabia-population/.

[10] Al Ghobain M, Alhamad EH, Alorainy HS, Al Kassimi F, Lababidi H, Al-Hajjaj MS. The prevalence of chronic obstructive pulmonary disease in Riyadh, Saudi Arabia: a BOLD study. Int J Tuberc Lung Dis. 2015;19(10):1252–7. doi: 10.5588/ijtld.14.0939 26459542

[11] Al Moamary MS, Al Ghobain MA, Al Shehri SN, Alfayez AI, Gasmelseed AY, Al-Hajjaj MS. The prevalence and characteristics of water-pipe smoking among high school students in Saudi Arabia. J Infect Public Health. 2012;5(2):159–68. doi: 10.1016/j.jiph.2012.01.002 22541263

[12] Al Moamary MS, Al Ghobain MO, Al Shehri SN, Gasmelseed AY, Al-Hajjaj MS. Predicting tobacco use among high school students by using the global youth tobacco survey in Riyadh, Saudi Arabia. Ann Thorac Med. 2012;7(3):122–9. doi: 10.4103/1817-1737.98843 22924068 PMC3425042

[13] Moradi-Lakeh M, El Bcheraoui C, Tuffaha M, Daoud F, Al Saeedi M, Basulaiman M, et al. Tobacco consumption in the Kingdom of Saudi Arabia, 2013: findings from a national survey. BMC Public Health. 2015;15:611-. doi: 10.1186/s12889-015-1902-3 26141062 PMC4491232

[14] El Hasnaoui A, Rashid N, Lahlou A, Salhi H, Doble A, Nejjari C. Chronic obstructive pulmonary disease in the adult population within the Middle East and North Africa region: rationale and design of the BREATHE study. Respiratory Medicine. 2012;106:S3–S15. doi: 10.1016/S0954-6111(12)70010-0 23290702

[15] Tageldin MA, Nafti S, Khan JA, Nejjari C, Beji M, Mahboub B, et al. Distribution of COPD-related symptoms in the Middle East and North Africa: results of the BREATHE study. Respir Med. 2012;106 Suppl 2:S25–32. doi: 10.1016/S0954-6111(12)70012-4 23290701

[16] Al Ghobain M, Al-Hajjaj MS, Wali SO. Prevalence of chronic obstructive pulmonary disease among smokers attending primary healthcare clinics in Saudi Arabia. Ann Saudi Med. 2011;31(2):129–33. doi: 10.4103/0256-4947.77485 21403413 PMC3102470

[17] Waness A, El-Sameed YA, Mahboub B, Noshi M, Al-Jahdali H, Vats M, et al. Respiratory disorders in the Middle East: a review. Respirology. 2011;16(5):755–66. doi: 10.1111/j.1440-1843.2011.01988.x 21564399

[18] Alsubaiei ME, Cafarella PA, Frith PA, McEvoy RD, Effing TW. Factors influencing management of chronic respiratory diseases in general and chronic obstructive pulmonary disease in particular in Saudi Arabia: An overview. Annals of thoracic medicine. 2018;13(3):144–9. doi: 10.4103/atm.ATM_293_17 30123332 PMC6073786

[19] Aldhahir AM, Alghamdi SM, Alqahtani JS, Alqahtani KA, Al Rajah AM, Alkhathlan BS, et al. Pulmonary rehabilitation for COPD: A narrative review and call for further implementation in Saudi Arabia. Annals of thoracic medicine. 2021;16(4):299–305. doi: 10.4103/atm.atm_639_20 34820017 PMC8588944

[20] Global Health Data Exchange 2019 [http://ghdx.healthdata.org/.

[21] Global, regional, and national incidence, prevalence, and years lived with disability for 328 diseases and injuries for 195 countries, 1990–2016: a systematic analysis for the Global Burden of Disease Study 2016. Lancet. 2017;390(10100):1211–59. doi: 10.1016/S0140-6736(17)32154-2 28919117 PMC5605509

[22] Global Burden of Disease Study 2019 (GBD 2019) Data Input Sources Tool 2019 [http://ghdx.healthdata.org/gbd-2019/data-input-sources?components=1&covariates=-1&locations=152.

[23] Global Burden of Disease Study 2019 (GBD 2019) Life Tables 1950–2019. 2019.

[24] Global, regional, and national disability-adjusted life-years (DALYs) for 333 diseases and injuries and healthy life expectancy (HALE) for 195 countries and territories, 1990–2016: a systematic analysis for the Global Burden of Disease Study 2016. Lancet. 2017;390(10100):1260–344. doi: 10.1016/S0140-6736(17)32130-X 28919118 PMC5605707

[25] Health. Mo. Statistics Year Book; 2014. [http://www.moh.gov.sa/Ministry/Statistics/Book/Pages/default.aspx.

[26] Al Ghobain M. The prevalence of chronic obstructive pulmonary disease in Saudi Arabia: Where do we stand? Annals of thoracic medicine. 2011;6(4):185–6. doi: 10.4103/1817-1737.84770 21977061 PMC3183633

[27] Quaderi SA, Hurst JR. The unmet global burden of COPD. Glob Health Epidemiol Genom. 2018;3:e4–e. doi: 10.1017/gheg.2018.1 29868229 PMC5921960

[28] Saudi Vision 2030 [https://www.vision2030.gov.sa/media/rc0b5oy1/saudi_vision203.pdf.

[29] Moradi-Lakeh M, El Bcheraoui C, Tuffaha M, Daoud F, Al Saeedi M, Basulaiman M, et al. The health of Saudi youths: current challenges and future opportunities. BMC Fam Pract. 2016;17:26. doi: 10.1186/s12875-016-0425-z 26946327 PMC4779578

[30] WHO. Noncommunicable diseases country profiles. Geneva 2018.

[31] Institute of Health Metrics and Evaluation. Better data for better health in the Kingdom of Saudi Arabia. [26/01/2022]. https://www.healthdata.org/ksa.

[32] Khan JH, Lababidi HMS, Al-Moamary MS, Zeitouni MO, Al-Jahdali HH, Al-Amoudi OS, et al. The Saudi Guidelines for the Diagnosis and Management of COPD. Annals of thoracic medicine. 2014;9(2):55–76. doi: 10.4103/1817-1737.128843 24791168 PMC4005164

[33] Al-Moamary MS, Köktūrk N, Idrees MM, Şen E, Juvelekian G, Saleh WA, et al. Unmet need in the management of chronic obstructive pulmonary disease in the Middle East and Africa region: An expert panel consensus. Respiratory Medicine. 2021;189:106641. doi: 10.1016/j.rmed.2021.106641 34649155

[34] Alqahtani JS, Oyelade T, Aldhahir AM, Alghamdi SM, Almehmadi M, Alqahtani AS, et al. Prevalence, Severity and Mortality associated with COPD and Smoking in patients with COVID-19: A Rapid Systematic Review and Meta-Analysis. PLoS One. 2020;15(5):e0233147. doi: 10.1371/journal.pone.0233147 32392262 PMC7213702

[35] WHO. Coronavirus disease (COVID-19) 2019 [https://www.who.int/emergencies/diseases/novel-coronavirus-2019.

[36] Alqahtani JS, Oyelade T, Aldhahir AM, Mendes RG, Alghamdi SM, Miravitlles M, et al. Reduction in hospitalised COPD exacerbations during COVID-19: A systematic review and meta-analysis. PLoS One. 2021;16(8):e0255659. doi: 10.1371/journal.pone.0255659 34343205 PMC8330941

[37] The burden of disease in Saudi Arabia 1990–2017: results from the Global Burden of Disease Study 2017. Lancet Planet Health. 2020;4(5):e195–e208. doi: 10.1016/S2542-5196(20)30075-9 32442495 PMC7653403

[38] Stevens GA, Alkema L, Black RE, Boerma JT, Collins GS, Ezzati M, et al. Guidelines for Accurate and Transparent Health Estimates Reporting: the GATHER statement. PLoS Med. 2016;13(6):e1002056. doi: 10.1371/journal.pmed.1002056 27351744 PMC4924581

